# A Novel Model for Instance Segmentation and Quantification of Bridge Surface Cracks—The YOLOv8-AFPN-MPD-IoU

**DOI:** 10.3390/s24134288

**Published:** 2024-07-01

**Authors:** Chenqin Xiong, Tarek Zayed, Xingyu Jiang, Ghasan Alfalah, Eslam Mohammed Abelkader

**Affiliations:** 1 Department of Building and Real Estate, Faculty of Construction and Environment, The Hong Kong Polytechnic University, Kowloon, Hong Kong 999077, China; chenqin.xiong@connect.polyu.hk (C.X.); tarek.zayed@polyu.edu.hk (T.Z.); 2 College of Mechanical and Electrical Engineering, Northeast Forestry University, Harbin 150040, China; jiangxy@spacestar.com.cn; 3 Department of Architecture and Building Science, College of Architecture and Planning, King Saud University, Riyadh 145111, Saudi Arabia; galfalah@ksu.edu.sa; 4 Structural Engineering Department, Faculty of Engineering, Cairo University, Giza 12613, Egypt

**Keywords:** surface cracks, bridges, YOLOv8s-Seg, asymptotic feature pyramid network, minimum point distance, middle aisle transformation

## Abstract

Surface cracks are alluded to as one of the early signs of potential damage to infrastructures. In the same vein, their detection is an imperative task to preserve the structural health and safety of bridges. Human-based visual inspection is acknowledged as the most prevalent means of assessing infrastructures’ performance conditions. Nonetheless, it is unreliable, tedious, hazardous, and labor-intensive. This state of affairs calls for the development of a novel YOLOv8-AFPN-MPD-IoU model for instance segmentation and quantification of bridge surface cracks. Firstly, YOLOv8s-Seg is selected as the backbone network to carry out instance segmentation. In addition, an asymptotic feature pyramid network (AFPN) is incorporated to ameliorate feature fusion and overall performance. Thirdly, the minimum point distance (MPD) is introduced as a loss function as a way to better explore the geometric features of surface cracks. Finally, the middle aisle transformation is amalgamated with Euclidean distance to compute the length and width of segmented cracks. Analytical comparisons reveal that this developed deep learning network surpasses several contemporary models, including YOLOv8n, YOLOv8s, YOLOv8m, YOLOv8l, and Mask-RCNN. The YOLOv8s + AFPN + MPDIoU model attains a precision rate of 90.7%, a recall of 70.4%, an F1-score of 79.27%, mAP50 of 75.3%, and mAP75 of 74.80%. In contrast to alternative models, our proposed approach exhibits enhancements across performance metrics, with the F1-score, mAP50, and mAP75 increasing by a minimum of 0.46%, 1.3%, and 1.4%, respectively. The margin of error in the measurement model calculations is maintained at or below 5%. Therefore, the developed model can serve as a useful tool for the accurate characterization and quantification of different types of bridge surface cracks.

## 1. Introduction

Hong Kong has emerged as one of the most economically advanced regions in the past five decades due to its unique cultural heritage and strategic location. This remarkable progress has been accompanied by significant improvements in urban infrastructure and facilities, including the construction of bridges and roads [1]. While urban development has undeniably brought convenience to the lives of citizens, it has also given rise to a pressing issue—the maintenance of bridges. In our daily lives, numerous factors, such as cyclic loading, fatigue stresses, and adverse long-term environmental conditions, have hastened the deterioration of bridge surfaces, resulting in problems like cracks, ruts, and potholes [2]. Among these concerns, cracks hold particular significance due to their direct impact on concrete structures’ safety, functionality, and durability [3]. Cracks provide an entry point for corrosive chemicals within the concrete, allowing water and de-icing salts to penetrate bridge decks, potentially causing damage to superstructures and aesthetic elements [4]. Since cracks are a critical indicator of a structure’s condition, they play a pivotal role in structural health monitoring [5]. Consequently, conducting a rigorous study to assess the extent and severity of cracks is essential for evaluating the condition of bridges and maintaining a comprehensive database for long-term bridge inspections and analysis. As a result, numerous countries have established comprehensive maintenance plans for their bridges. For example, the United States mandates biennial bridge inspections in compliance with American Association of State Highway and Transportation Officials (AASHTO) requirements [6]. In the United Kingdom, bridge inspections occur every one to three years in accordance with established standards [7]. In China, specific standards dictate the detection and quantification of bridge cracks wider than 0.2 mm [8]. 

In practical terms, due to its simplicity, human visual inspection remains the predominant method for monitoring bridge health [9]. However, this method heavily relies on expertise, experience, and subjectivity, which can result in imprecise and unreliable assessment outcomes [10,11]. Furthermore, visual inspection is often impractical for inaccessible areas of a bridge, such as columns and intersections [12]. In such cases, bridge inspectors resort to deploying inspection vehicles as manual operation platforms to measure the size of bridge cracks. Nevertheless, this approach has its own set of limitations, including traffic disruptions, time consumption, and high maintenance costs [13]. The advent of computer vision models has paved the way for automatic inspection technologies, offering enhanced efficiency and precision in contrast to manual measurements, which are susceptible to inaccuracies and subjectivity [14,15]. 

However, the application of these technologies presents various challenges and limitations. Firstly, many studies in this field focus on crack detection under ideal conditions, often on surfaces consisting entirely of concrete or asphalt. In real-world scenarios, crack detection tasks frequently entail complex backgrounds with diverse elements, such as tree leaves and varying lighting conditions, rendering the accurate distinction of cracks challenging [16]. Furthermore, traditional bridge distress inspection commonly involves image processing techniques employing edge detection and image thresholding. Cracks are identified based on changes in edge gradients derived from the intensity difference relative to the background and are extracted through threshold segmentation [17]. However, this method is significantly affected by environmental factors during image collection, including variations in lighting conditions and the presence of oil stains [3]. The current study introduces an integrated framework tailored explicitly for the segmentation of road cracks and the quantification of surface features, with a particular emphasis on addressing the challenges posed by complex backgrounds. In contrast to existing research in the same domain, our study presents several noteworthy contributions:

(1) We provide a comprehensive literature review that delves into the realms of bridge crack detection and segmentation, offering a thorough understanding of the existing body of knowledge in this field.

(2) To enrich the research landscape, we have meticulously curated a new bridge crack dataset customized to the distinctive context of Hong Kong. This dataset encompasses a diverse array of crack samples, faithfully representing the complexities encountered in real-world scenarios, including various crack sizes and intricate crack backgrounds.

(3) Our study pioneers the development of a novel YOLOv8-AFPN-MPD-IoU, an advanced approach facilitating precise and timely detection and segmentation of cracks within Hong Kong’s bridges with complex backgrounds, thus enhancing process efficiency. The AFPN is incorporated into the YOLOv8 neck, effectively reducing information gaps between non-adjacent layers and facilitating improved feature fusion. The MPD-IoU (minimum point distance intersection over union) loss function is deployed to solve the problem that the predicted bounding box possesses the same aspect ratio as the ground-truth bounding. In terms of recognition precision, model size, and reference times, the proposed model emerges as the optimal choice for deploying portable devices in practical applications.

(4) Furthermore, we employ a distance transform method (DTM) to accurately measure the length and width of segmented road cracks at the pixel level. This approach enhances precision and minimizes the impact of varying lighting conditions, a crucial aspect of our methodology.

## 2. Literature Review 

Crack identification is mainly divided into traditional methods based on digital image processing and methods based on deep learning. Digital image processing methods based on edge detection and threshold segmentation are highly susceptible to ambient environmental conditions and image quality requirements. Additionally, these techniques may fail when encountered with real-world scenarios such as tree leaves and varying lighting conditions [18]. Likewise, they remain highly dependent on the design of feature descriptors, and therefore, they are difficult to be generalized [19,20,21]. In this respect, some previous research attempts relied on the use of threshold segmentation [22], Otsu [23], improved Otsu [24,25,26], edge detectors [27,28], improved watersheds [29], artificial neural networks [30,31], hybrid artificial neural networks [32], and support vector machines [33,34,35] for crack detection and assessment. 

Aside from data dependence, deep learning addresses the limitations inherent in the typical machine learning models mentioned above. The advancement of graphical processing units (GPUs) has significantly accelerated image processing on computers. Consequently, deep learning techniques can now be effectively employed for tasks such as image object detection and segmentation, including the management of building construction [36]. Convolutional neural networks (CNNs) are among the popular techniques used to extract category and location information from images in object detection tasks. However, the process of generating region proposals using selective search remains slow and exhibits limited accuracy for two-stage detectors [37]. Rosso et al. [38] presented a ResNet-50-based framework for defect classification in ground penetrating radar profiles. The authors implemented bi-dimensional Fourier transform as a preprocessing convolution operation, and vision transformer architecture was incorporated, resulting in better localization of defects. In another research effort, Park et al. [39] introduced a machine-learning-based model for predicting depths of concrete cracks in thermal images. The four machine learning models included AdaBoost, gradient boosting, random forest, and multilayer perceptron. The AdaBoost model demonstrated the highest prediction accuracy with a determination coefficient and mean absolute percentage error of 98.96% and 0.6%, respectively. They also showed that the combination of principal component analysis with AdaBoost or gradient could properly detect microcracks. 

Among the bounding box detection models, Zhang et al. [40] created a single-stage detector based on you only look once (YOLOv3) to locate bridge surface defects like cracks, spalling, pop-outs, and exposed rebar. In another study, Deng et al. [41] utilized YOLOv2 to locate cracks in real-world images contaminated with handwriting scripts. In a third study, Teng et al. [42] investigated the use of 11 pre-trained deep learning networks to be leveraged as feature extractors of YOLOv2. Among them, there were Alex Net, VGG16, VGG19, ResNet50, ResNet101, Google Net, etc. They deduced that the integration of ResNet18 and YOLOv2 was deemed appropriate in terms of computational accuracy and efficiency. Similarly, Qiu and Lau [43] elucidated that ResNet50-based YOLOv2 and YOLOv4-tiny demonstrate exceptional accuracy, fast processing speed, and notable proficiency in detecting small cracks in tiled sidewalks for crack detection. Xiang et al. [44] addressed various challenges encountered by UAV-based crack detection networks, including low efficiency and accuracy stemming from road noise such as shadows, occlusions, and low contrast. Consequently, the YOLO architecture is recognized for its capability to identify cracks amidst complex backgrounds.

However, the bounding box outcomes provided by crack recognition lack the potential for further quantitative analysis, thereby rendering crack recognition less suitable as a crack measurement algorithm [11]. The task of object segmentation evolves from object detection, requiring a comprehensive understanding of the image scene at the pixel level. In crack segmentation tasks, the input image typically contains only cracks and background. The resulting output consists of binary values of 1 and 0, representing crack pixels and background pixels, respectively [45]. As a result, the outcomes of crack segmentation can facilitate subsequent geometric calculations of crack length and width. The human brain rapidly processes distinct shapes and irregular contours present in objects, similar to an objective segmentation task. Consequently, creating a detection frame outside the target object is deemed unnecessary and incongruent with human visual interpretations [46]. Therefore, the utilization of the YOLOv8-seg model in bridge inspection endeavors represents a promising initiative.

## 3. Research Methodology 

**Figure 1:**
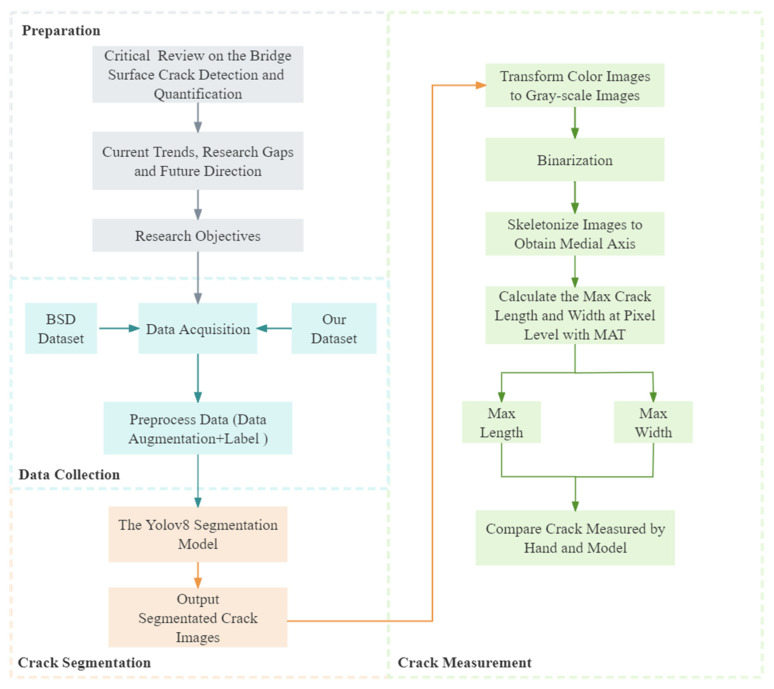
Framework of the developed YOLOv8-APF-MPDIOU model for crack instance segmentation.

This research study comprises three distinct phases within its framework: a systematic review, data collection, and crack measurement. Illustrated in Figure 1, this framework delineates a structured approach to bridge crack segmentation and measurement. The model developed is grounded in a conceptualization revolving around three essential modules. The initial module involves a comprehensive examination of existing models for detecting and quantifying surface cracks on bridge decks. This critical review not only identified research gaps, future research directions, and clear objectives but also prompted the utilization of a publicly available database to augment the diversity and complexity of image scenarios used in experimental settings. However, currently, the images provided lack sufficient power to fully capture the complex diversity of real-world scenes. Therefore, a set of supplementary images featuring various bridge defects was curated specifically for this purpose, utilizing data augmentation—an extensively employed method to mitigate overfitting in deep learning models while enhancing their diversity and generalizability [47]. The images were captured at various locations across Hong Kong, encompassing a diverse array of environmental factors. These include variations in sunlight conditions, diverse surface textures, different bridge locations, instances of graffiti, and occurrences of water damage. Various transformations, such as lighting adjustments, random element addition, noise introduction, flipping, and image bounding box manipulation, were applied to augment the Hong Kong dataset. At this juncture, the integrated dataset combines the publicly accessible benchmark dataset of bridge surface cracks (BSD) sourced from [48] alongside the Hong Kong bridge crack dataset (HKBCD). The third module of this study focuses on developing a novel YOLOv8-AFPN-MPDIoU model for surface crack segmentation, complemented by the utilization of the distance transform method (DTM) for precise calculation of bridge crack width and length.

## 4. Model Development 

This section delineates the basic components of the developed YOLOv8-AFPN-MPDIoU model. 

### 4.1. Basics of YOLOv8-Segmentation 

**Figure 2:**
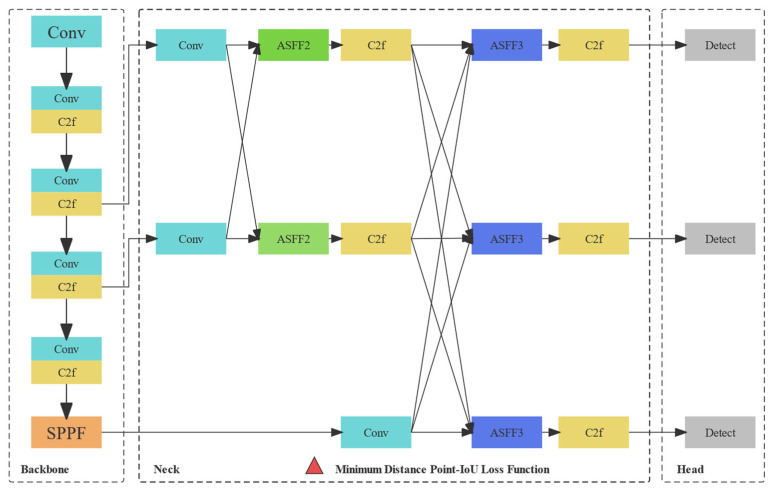
Architecture of the developed YOLOv8-AFPN-MPDIoU model.

**Figure 3:**
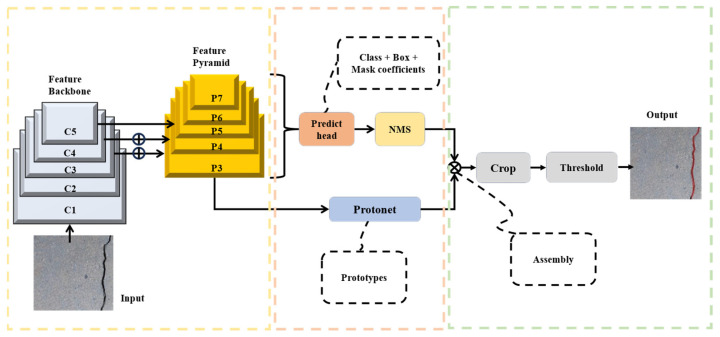
Structure of YOLACT network.

Architecture of the developed YOLOv8-AFPN-MPDIoU model is depicted in Figure 2. Its distinctive features lie in the following:

1. The YOLOv8 network offers support for object detection, tracking, and various additional tasks, including instance segmentation, image classification, and key-point detection. Similar to YOLOv5, YOLOv8 presents five distinct scales of models (n, s, m, l, x) with increasing depth and width from left to right [49].
2. In alignment with the efficient layer aggregation network (ELAN) design philosophy, YOLOv8 replaces the C3 structure within the YOLOv5 backbone network with a C2f structure. This modification enables YOLOv8 to retain its lightweight characteristics while enhancing the flow of gradients [50]. In comparison to YOLOv5, YOLOv8 demonstrates more pronounced disparities in its head section due to the integration of the widely adopted decoupled head structure.
3. YOLOv8 adopts the Task-Aligned-Assigner strategy for positive sample assignment in loss function calculation [51]. Additionally, it introduces the distribution focal loss (DFL). During training, the strategy of disabling mosaic augmentation in the final ten epochs is incorporated, as inspired by YOLOX, to effectively enhance precision in the data augmentation process.
4. YOLOv8s-Seg is an extension of the YOLOv8 object detection model specifically tailored for performing segmentation tasks. This network draws upon the principles of the YOLACT network to achieve real-time instance segmentation of objects while maintaining a high segment mean average precision [52]. The structural overview of the YOLACT network is presented in Figure 3.
5. The YOLOv8-Seg network, version ultralytics 8.0.201, comprises three primary components: the backbone, the neck, and the head. In the associated GitHub repository, five distinct scale models of the network are available, specifically YOLOv8n-Seg, YOLOv8s-Seg, YOLOv8m-Seg, YOLOv8l-Seg, and YOLOv8x-Seg. In this study, experiments were conducted using YOLOv8-Seg models at various scales to assess the segment mAP50 and model size. Given the minimal presence of cracks in each image, the first four scales of models were utilized to identify the most suitable scale.

### 4.2. Asymptotic Feature Pyramid Network

In tasks related to object detection and segmentation, the multi-scale feature extraction process plays a key role in object encoding and scale change processing. A common approach is to utilize well-established top-down or bottom-up feature pyramid networks. However, these methods are susceptible to the problem of feature information loss and deterioration, which in turn adversely affects the integration of information between two non-adjacent layers. Within the prevailing architectures of feature pyramids, the challenge lies in the necessity for high-level features located at the pinnacle of the pyramid to traverse multiple intermediate scales and engage with features at these intermediate levels before finally fusing with the low-level features at the base. Throughout this process of propagation and interaction, there is a risk of the semantic information contained within the high-level features being lost or compromised. In contrast, the bottom-up pathway of the PAFPN (pyramid attention feature pyramid network) introduces a converse challenge: the detailed information originating from the lower-level features could potentially suffer loss or deterioration during the process of propagation and interaction.

#### 4.2.1. Asymptotic Architecture 

**Figure 4:**
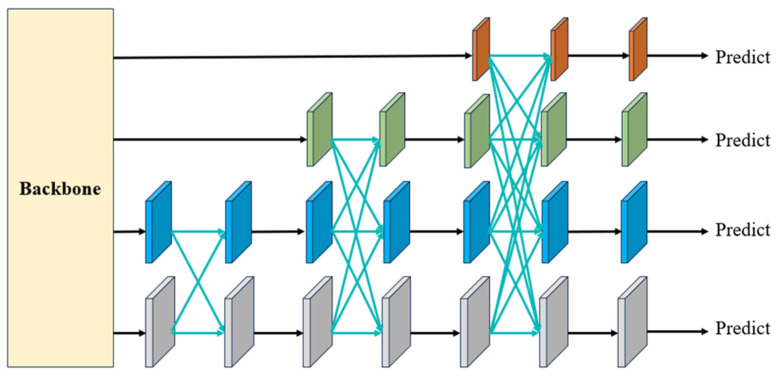
Architecture of the proposed asymptotic feature pyramid network.

To address this challenge, the asymptotic feature pyramid network (AFPN) was developed by Yang et al. [53]. The issue stems from the substantial semantic gap between non-adjacent hierarchical features, which is especially pronounced between the lowest- and highest-level features. This gap hinders the effective fusion of non-adjacent hierarchical features. However, the AFPN architecture is designed to be asymptotic, resulting in a closer alignment of semantic information across different hierarchical feature levels during the asymptotic fusion process. This, in turn, mitigates the aforementioned issues. As illustrated in Figure 4, during the bottom-up feature extraction process of the backbone network, the AFPN gradually integrates low-level features, followed by intermediate-level features, before culminating in the fusion with the highest-level feature, which is the most abstract. Black arrows denote convolutions, while blue arrows indicate adaptive spatial fusion. In this proposed model, the AFPN is incorporated into the YOLOv8 neck, effectively reducing information gaps between non-adjacent layers and facilitating improved feature fusion. 

#### 4.2.2. Adaptive Spatial Fusion

**Figure 5:**
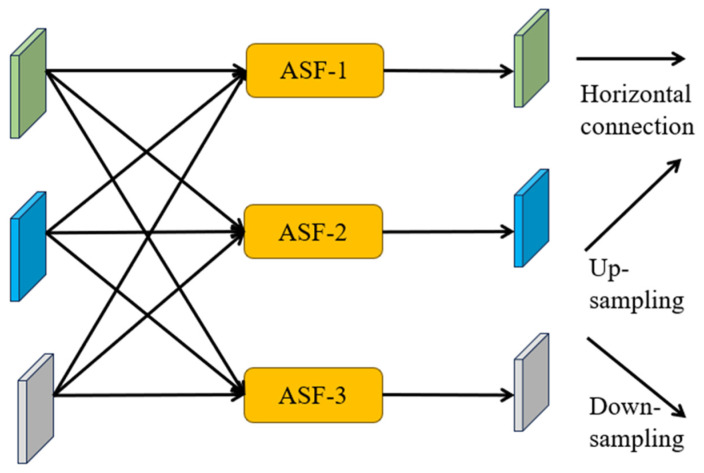
Adaptive spatial fusion operation.

The ASFF technique is utilized to assign variable spatial weights to different feature levels during multi-level feature fusion, enhancing the importance of critical levels while minimizing the impact of conflicting information from diverse objects. Illustrated in Figure 5, features from three different levels are combined. This Figure serves as an illustration of feature fusion at three different levels, but we can adapt the method as needed for cases with more or fewer levels. Representing the feature vector transitioning from level *n* to level *l* at position (i,j) as $x_{ij}^{n\to l}$, the resultant feature vector $y_{ij}^{l}$ is attained through adaptive spatial fusion, formally defined as the linear combination of feature vectors $x_{ij}^{1\to l}$, $x_{ij}^{2\to l}$, and $x_{ij}^{3\to l}$ (refer to Equation (1)).

(1) $$y_{ij}^{l}=\alpha_{ij}^{l}\cdot x_{ij}^{1\to l}+\beta_{ij}^{l}\cdot x_{ij}^{2\to l}+\gamma_{ij}^{l}\cdot x_{ij}^{3\to l}$$

At level $\alpha_{ij}^{l}$, $\beta_{ij}^{l}$ and $\gamma_{ij}^{l}$ represent the spatial weights of the features across the three levels, with the constraint $\alpha_{ij}^{l}+\beta_{ij}^{l}+\gamma_{ij}^{l}=1$. Due to variations in the number of fused features at each stage of the AFPN, we introduce stage-specific adaptive spatial fusion modules [54]. The adaptive spatial fusion operation is shown in Figure 5. 

### 4.3. Minimum Point Distance-IoU Loss Function

The optimization process of the YOLOv8 model occurs across two dimensions: classification and regression. While the classification loss continues to employ binary cross-entropy loss (BCEL), the regression component integrates focal distribution loss (DFL) and bounding box regression loss (BBRL). Equation (2) comprehensively represents the loss function:

(2) $$f_{\mathrm{Loss}}=\lambda_{1}f_{BCEL}+\lambda_{2}f_{DFL}+\lambda_{3}f_{BBRL}$$

Here, $f_{\mathrm{Loss}}$ denotes the total loss; $\lambda_{1}$, $\lambda_{2}$, and $\lambda_{3}$ represent the weighting factors assigned to each loss term; and $f_{BCEL}$, $f_{DFL},$ and $f_{BBRL}$ are individual loss functions for binary cross-entropy, focal distribution loss, and bounding box regression loss, respectively. BCEL loss is utilized for classification, assessing the dissimilarity between predicted class probabilities and ground-truth labels. DFL is applied for regression, considering the distribution of predicted bounding box values and focusing on challenging samples. Meanwhile, BBRL aims to minimize the difference between predicted and ground-truth bounding box coordinates.

Bounding box regression (BBR) has established itself as a crucial component in object detection and instance segmentation, serving as a pivotal step in precise object localization. Nevertheless, a substantial limitation of most prevailing loss functions for bounding box regression lies in their inability to be effectively optimized when the predicted bounding box possesses the same aspect ratio as the ground-truth bounding box but exhibits distinct width and height values. To address these aforementioned challenges, we conducted a comprehensive exploration of the geometric attributes associated with horizontal rectangles. Consequently, we introduce a novel bounding box similarity comparison metric, denoted as MPD-IoU (minimum point distance intersection over union), which encapsulates all the relevant factors considered in existing loss functions. This includes considerations such as the overlapping or non-overlapping area, the distance between central points, and variations in width and height, while concurrently simplifying the computational process.

**Figure 6:**
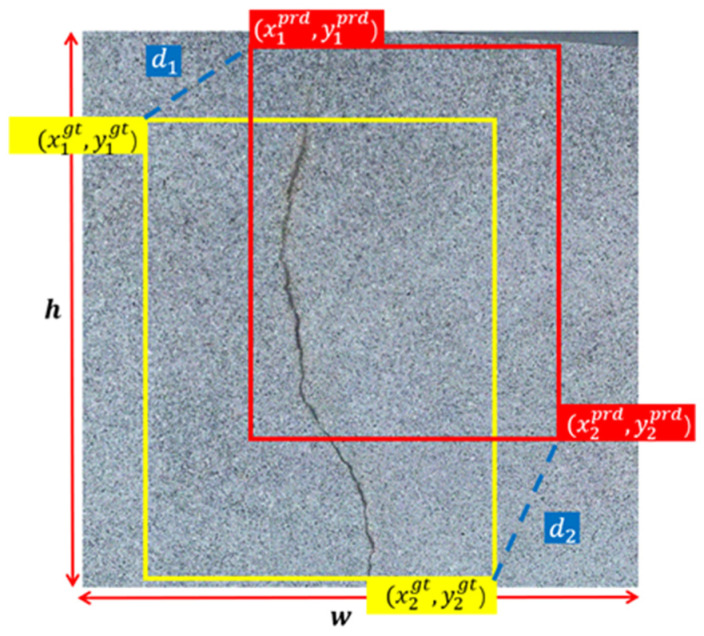
Visual illustration of the proposed $L_{\mathrm{MPDIoU}}$.

**Figure 7:**
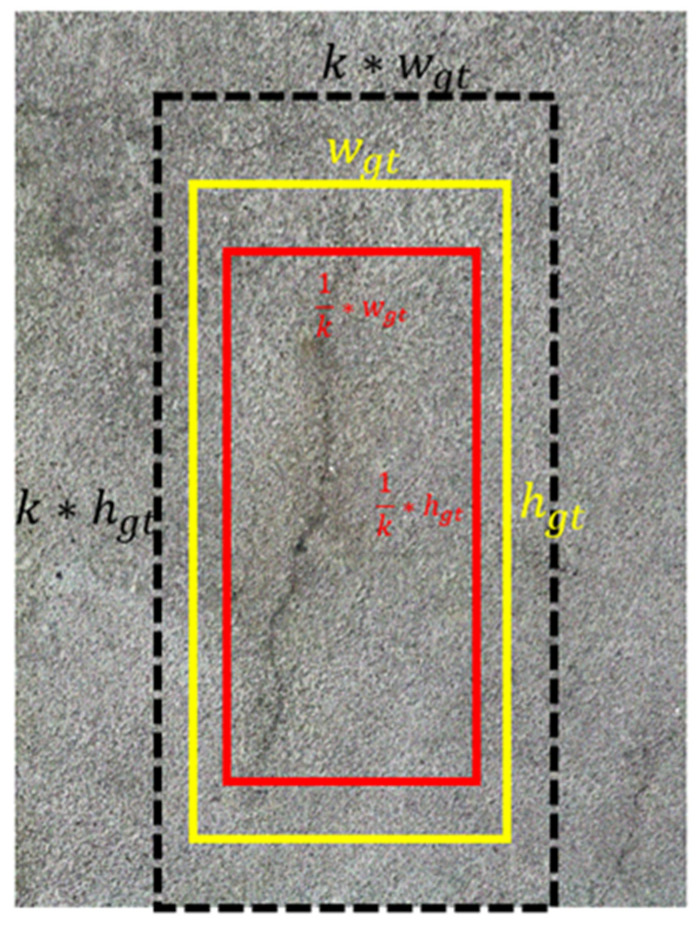
Visual presentation of predicted bounding boxes and ground-truth bounding boxes.

In contrast to the complete intersection over union loss function (C-IoU loss function) employed in YOLOv8, the minimum point distance-IoU loss function (*MPD*-*IoU* loss function) is employed for comparison against other loss functions based on IoU [55]. Figure 6 demonstrates a visual presentation of the proposed MPD-IoU loss function. Figure 7 provides visual examples of predicted bounding boxes and ground-truth bounding boxes. They share the same aspect ratio but differ in width and height, where k > 1 and k ∈ R. In these visualizations, the yellow box signifies the ground-truth bounding box, while the red box and black box represent the predicted bounding boxes. Mathematically, the key components of the *MPD*-*IoU* metric are defined using Equations (3)–(8).

(3) $$d_{1}^{2}={\left(x_{1}^{prd}-x_{1}^{gt}\right)}^{2}+{\left(y_{1}^{prd}-y_{1}^{gt}\right)}^{2}$$

(4) $$d_{2}^{2}={\left(x_{2}^{prd}-x_{2}^{gt}\right)}^{2}+{\left(y_{2}^{prd}-y_{2}^{gt}\right)}^{2}$$

(5) $$w_{gt}=x_{2}^{gt}-x_{1}^{gt},h_{gt}=y_{2}^{gt}-y_{1}^{gt},w_{prd}=x_{2}^{prd}-x_{1}^{prd},h_{prd}=y_{2}^{prd}-y_{1}^{prd}$$

(6) $$MPD-IoU=IoU-\frac{d_{1}^{2}}{h_{gt}^{2}+w_{gt}^{2}}-\frac{d_{2}^{2}}{h_{prd}^{2}+w_{prd}^{2}}$$

(7) $$L_{MPDIoU}=1-MPD-IoU$$

(8) $$f_{Loss}=\lambda_{1}f_{BCEL}+\lambda_{2}f_{DFL}+\lambda_{3}f_{MPD-IoU}$$

where, the overall loss function, denoted as $f_{Loss}$, is assigned weights (*λ*₁, *λ*₂, *λ*₃) to balance the contributions of binary cross-entropy loss $f_{BCEL}$, distance-based focal loss ($f_{DFL}$), and the proposed MPD-IoU-based loss ($f_{MPD-IoU}$). Here, ($x_{c}^{gt}$, $y_{c}^{gt}$) and ($x_{c}^{prd}$, $y_{c}^{prd}$) denote the coordinates of the central points of the ground-truth bounding box and the predicted bounding box, respectively. Similarly, $w_{gt}$ and $h_{gt}$ represent the width and height of the ground-truth bounding box, while $w_{prd}$ and $h_{prd}$ denote the width and height of the predicted bounding box. 

The expressiveness of our MPD-IoU metric is underscored by the fact that all the factors considered in existing loss functions can be determined by the coordinates of the top-left and bottom-right points, encompassing the non-overlapping area, central point distance, and deviations in width and height. As a result, our proposed MPD-IoU metric not only offers a comprehensive consideration of these factors but also streamlines the calculation process. It is worth noting that when the aspect ratio of the predicted bounding box matches that of the ground-truth bounding box, the MPD-IoU metric ensures that the predicted bounding box’s value is lower when it is contained within the ground-truth bounding box, compared to cases where the predicted bounding box extends beyond the boundaries of the ground-truth bounding box. This unique characteristic enhances the precision of bounding box regression, ultimately reducing redundancy in the predicted bounding boxes.

### 4.4. Crack Skeleton Extraction and Measurement

**Figure 8:**
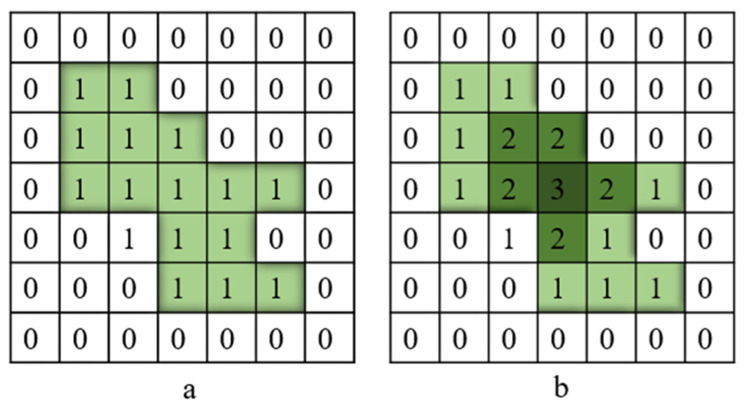
Representation of the incorporated distance transform. (**a**) Before applying distance transform and (**b**) After applying distance transform.

The extraction of crack skeletons serves as a crucial step in deriving geometric properties from segmented crack images, encompassing measurements such as length, width, and area. In this investigation, we employ the medial axis transformation (MAT) method to delineate the outline of the target crack based on binary images [56]. This process involves selecting a point $P_{t}$ within the interior region (*A*) of any crack and identifying the point ($P_{t}^{\prime }$) on the crack boundary ($A^{C}$) that is closest to $P_{t}$. If multiple $P_{t}^{\prime }$ points are found, $P_{t}$ is designated as the skeleton point of the crack. The search for $P_{t}^{\prime }$ is formalized in Equation (9):

(9) $$d_{t}\left(P_{t},A^{C}\right)=inf\left\{d\left(P_{t},z\right)|z\in A^{C}\right\}$$

where inf denotes the lower bound, $d_{t}$ and d represent Euclidean distances, and *z* represents any point on $A^{C}$. Points with multiple neighbors are excluded from consideration. Subsequently, a distance transformation is executed from all foreground pixels to the background, with “1” representing the foreground and “0” denoting the background. This process is illustrated in Figure 8. Finally, the target is reduced to a skeleton based on the outcomes of the distance transformation. Essentially, this process mirrors the boundary erosion process.

**Figure 9:**
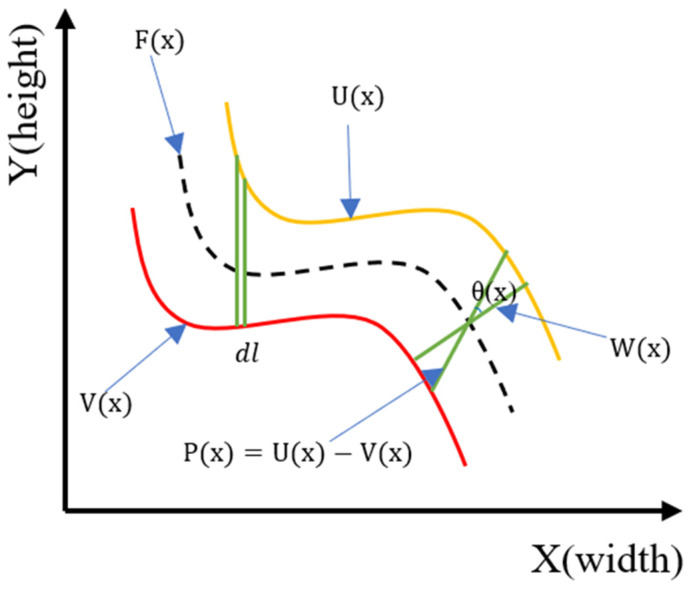
Schematic diagram of calculation method of crack length, width.

To describe MAT clearly, a diagram is used to explain how the algorithm calculates crack width and length. The coordinate system is shown in Figure 9. Each pixel in the crack image is our coordinate [57]. Therefore, a pixel coordinate system is established with red and yellow line segments representing the boundaries of the crack. The black line segment in the middle is the crack skeleton, calculated based on the central skeleton algorithm, and *W*(*x*) is the calculated average width of the crack. Part is *dl*. Within the scope of this study, the width of each crack in the workpiece is represented by pixel values. Thus, each pixel represents the width or length, making it easy to calculate the number of pixels contained in the crack. The calculation formula for determining the crack length (*L*) is shown in Formula (10).

(10) $$L=\int_{c}\;f\left(x,y\right)dl≅\sum f\left(x,y\right)dl$$

The function $f\left(x,y\right)$ acts as a geometric calibration index, aligning the detected displacements of points within the image. Simultaneously, dl denotes the finite length of each skeleton unit. c represents the path of the crack skeleton. The crack skeleton is a central line that traces the main path of the crack through the image. This skeleton is derived from the central skeleton algorithm, which identifies the midline of the crack. Integration or summation along the crack skeleton accumulates the lengths of small segments dl corrected by $f\left(x,y\right)$.

The pixel-level crack width value is computed by the pixel distance calculation between the crack pixel (1) and the intact pixel (0) using the Euclidean distance transform (EDT) algorithm. The Euclidean distance (*d*) of the two pixels, $P_{t}$ and $P_{t}^{\prime }$, is expressed by Equation (11) [58].

(11) $$(P_{t},P_{t}^{\prime })=\sqrt{{\left(x_{P_{t}}-x_{P_{t}^{\prime }}\right)}^{2}+{\left(y_{P_{t}}-y_{P_{t}^{\prime }}\right)}^{2}}\left(P_{t}\in A,P_{t}^{\prime }\in A^{C}\right)$$

where *t* is an indexing variable used to uniquely identify each pixel within the respective sets A and $A^{C}$ to calculate the Euclidean distance between a cracked pixel and an intact pixel. A is the set of green pixels represented by 1 in Figure 8, and $A^{C}$ is the white pixel, complement of the green pixel set, A.

### 4.5. Performance Evaluation 

The selection of suitable evaluation metrics holds paramount importance when assessing various segmentation models. While accuracy remains a prominent benchmark for existing instance segmentation models, the advent of real-time and lightweight models has introduced new considerations for practical deployment on equipment. Consequently, our proposed model shall undergo evaluation with a multifaceted approach, scrutinizing not only its accuracy but also its runtime performance and model complexity to ascertain its suitability for practical applications.

#### 4.5.1. Accuracy

In this study, we conducted a comprehensive evaluation of the enhanced YOLOv8s-Seg model, employing a range of performance metrics, including precision, recall, *F*1-score, and segment mAP at both 50% and 75% intersection over union (IoU) thresholds [59,60,61]. Our assessment focused on two distinct aspects: the accuracy of crack detection, which was quantified using precision, recall, and *F*1-score, and the quality of the segmentation results, which were appraised through segment mAP.

(12) $$\mathrm{precision}=\frac{TP}{\left(TP+FP\right)}\times 100\%$$

(13) $$\mathrm{recall}=\frac{TP}{\left(TP+FN\right)}\times 100\%$$

(14) $$F1=2\times \mathrm{precision}\times \frac{\mathrm{recall}}{\mathrm{precision}+\mathrm{recall}}$$

(15) $${seg}_{mAP}=\sum_{i=1}^{c}\;\frac{AP(i)}{C}$$

Here, *TP* represents true positive samples, which are actual positive instances correctly predicted as such. *FP* indicates false positive samples, signifying actual negative instances erroneously identified as positive, while *FN* stands for false negatives, representing actual positive instances wrongly classified as negative. *AP*(*i*) corresponds to the average precision for each segmentation category, and *C* represents the total number of segmentation categories. Notably, the segmentation model’s performance is directly linked to the cumulative *AP* score, and a higher *AP* score is indicative of improved segmentation quality.

#### 4.5.2. Computational Time 

In the realm of model evaluation, two critical indicators to gauge efficiency are training times and inference times. Training time denotes the duration required for a model to converge during the training process, while inference time signifies the time taken to process an image, thereby reflecting the model’s suitability for practical industrial applications. To streamline the assessment process, the use of FPS (frames per second) has become a prevalent metric for evaluating the efficiency of instance segmentation methods. This metric offers a straightforward means for researchers to identify and select faster inference models when operating under comparable conditions [62,63].

#### 4.5.3. Model Complexity

Model complexity plays a crucial role in evaluating the practical usability of a model, as it directly influences storage requirements and computational demands. In our study, it specifically refers to the storage needs linked to model parameters, a factor closely tied to the implementation of algorithms on mobile devices.

## 5. Model Implementation 

This section addresses data collection and analysis of bridge surface cracks. 

### 5.1. Data Collection 

**Figure 10:**
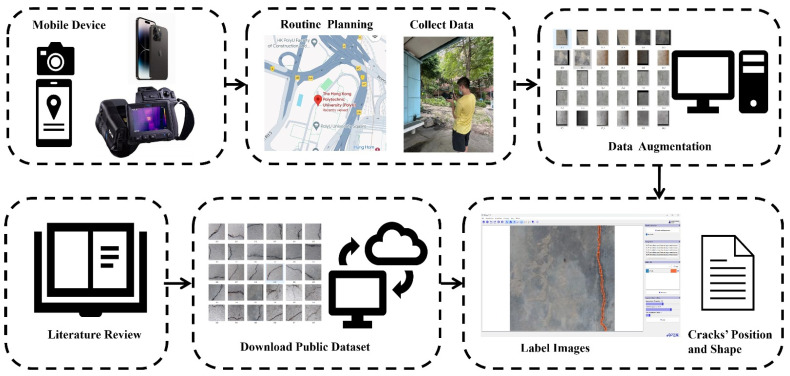
The procedures of crack image capture and preprocessing.

The model training was conducted on a high-performance computing workstation equipped with specific hardware, including GPUs (GeForce RTX 3090, NVIDIA, Santa Clara, CA, USA), a CPU (Intel(R) Core (TM) i9-10900 CPU @ 2.80 GHz, Intel, Santa Clara, CA, USA), and 22.6 GB of RAM. The system operated on Windows 10 and utilized PyTorch 2.0.0 as the deep learning framework, with the software environment incorporating CUDA 11.7 and Python 3.8. Detailed specifications of the hardware and software setups are provided in the accompanying table. The data collection and processing methodologies for this study are detailed in Figure 10. The dataset comprises a total of 1600 cases, meticulously annotated for accuracy. Among these, 240 images were sourced from Hong Kong bridges and their associated data enhancements, with the remaining 1360 images obtained from the publicly available database of BSD. The latter subset of 1360 bridge crack images, each sized 224 × 224 pixels, was randomly selected from the BSD dataset. It encompasses images with cracks of variable size and width, stains, shadows, complex backgrounds, and non-uniform illumination. The selected images contain instances of transverse cracks, diagonal cracks, and reticular cracks. 

**Figure 11:**
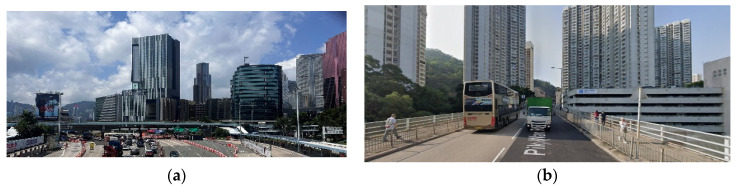
Sources of the HKBCD dataset. (**a**) Footbridge across the Hong Kong Polytechnic University; (**b**) flyover bridge at Pik Wan road.

**Figure 12:**
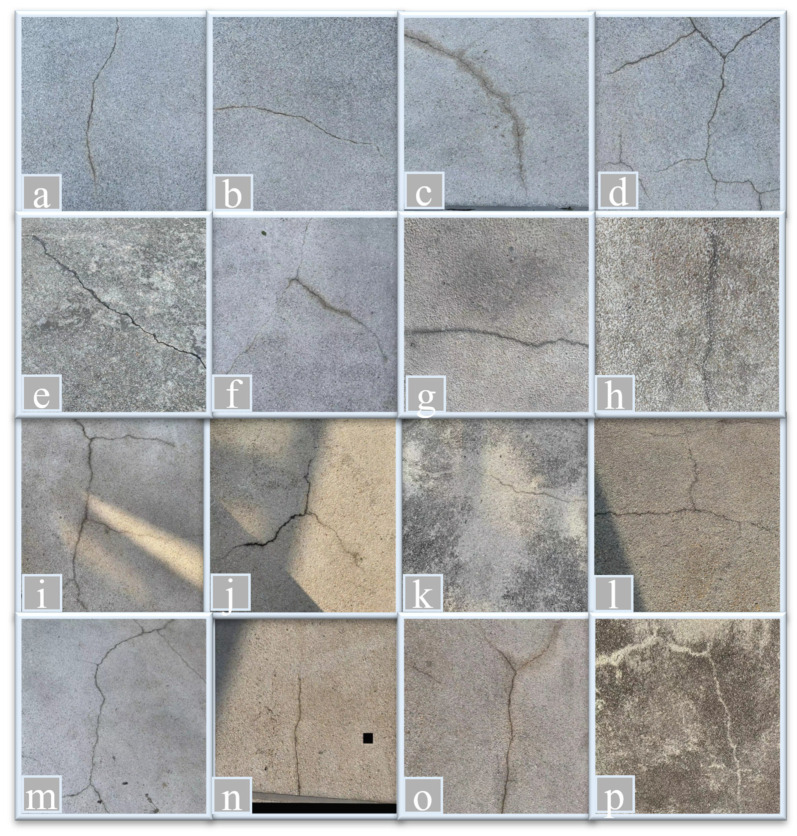
Sample of images from HKBCD datasets. (**a**–**d**) Different Types of cracks, (**e**–**h**) Different types of bridge materials, (**i**–**l**) Different lighting conditions and (**m**–**p**) Different types of noises.

Additionally, 60 crack images were captured from bridges near the Hong Kong Polytechnic University. The images in the Hong Kong bridge crack dataset (HKBCD) were obtained using an iPhone 14 Pro and a FLIR thermal camera capable of producing RGB images. The Hong Kong subset used in this study was collected from two distinct locations: the footbridge surrounding the Hong Kong Polytechnic University campus and the K74 flyover bridge at Pik Wan road, depicted in Figure 11. To enrich the diversity of the dataset, images were captured under a variety of conditions, encompassing different crack shapes, backgrounds, brightness levels, and noise settings. As illustrated in Figure 12, images (a)–(d) showcase four types of cracks: transversal, longitudinal, diagonal, and reticular. Images (e)–(h) depict cracks on different bridge materials, including concrete and asphalt. Images (i)–(l) display bridge cracks under varying light intensities. Images (m)-(p) are influenced by different types of noise, such as graffiti, water stains, moss, and other obstructions. By incorporating these variations, the dataset ensures a comprehensive representation of real-world scenarios, enhancing the robustness and generalizability of the analysis. The augmentation of the HKBCD added an additional 240 crack images, bringing the total compiled dataset to 1600 images. This dataset was then divided into training, validation, and test sets in a 7:1:2 ratio, resulting in 1120 training images, 160 validation images, and 320 test images for the crack segmentation task. Further details on the dataset partitioning are provided in Table 1. Notably, the dataset encompasses various types of concrete cracks, categorized as follows: approximately 20% of the images depict reticular cracks, 40% feature diagonal cracks, and the remaining 40% display linear cracks. Reticular cracks form a mesh-like pattern and are often attributed to concrete shrinkage or restrained thermal movements. Diagonal cracks appear diagonally across concrete surfaces and are commonly caused by structural overloading or differential settlement. Transverse cracks run perpendicular to the direction of tensile stress and are frequently associated with drying shrinkage, thermal contraction, or excessive applied loads.

**Figure 13:**
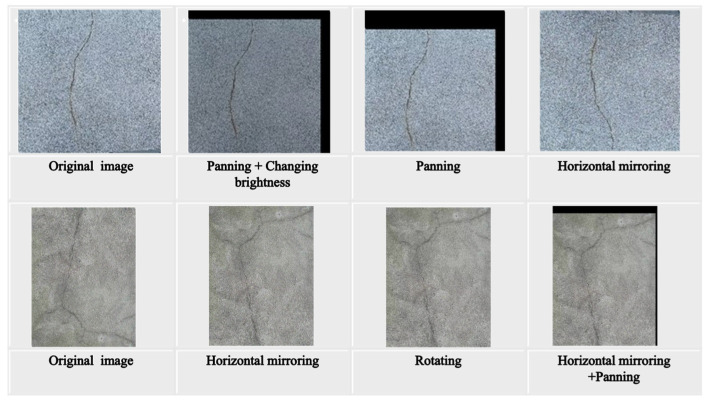
Samples of data augmentation, adjustments in brightness, panning, mirroring, and rotation.

Four data augmentation techniques were implemented to bolster the model’s generalization capacity, encompassing adjustments in brightness, panning, mirroring, and rotation. It is worth noting that the level of added noise was meticulously regulated to prevent adverse effects on the model’s output. Consequently, a total of 240 crack images were generated for the dataset. The EIseg software 1.1.1 was employed to annotate this dataset. The results of the data augmentation techniques are presented in Figure 13, illustrating four methods: brightness, panning, mirroring, and rotation. The developed YOLOv8-AFPN-MPD-IoU model underwent training using the following hyperparameters: a learning rate of 0.01, momentum of 0.937, weight decay of 0.0005, and a batch size of 8. A stochastic gradient descent (SGD) optimizer was utilized for training the model over 300 epochs in total. Hardware and software requirements for this research study are detailed in Table 2.

### 5.2. Performance Comparison of YOLOv8 Architectures 

In this research study, we conducted a series of experiments employing YOLOv8-Seg models at various scales with the objective of evaluating segment mAP50 and model size. Given the limited occurrence of cracks within each image, the initial four scales of models were examined to determine the most suitable scale. Similar to YOLOv5, YOLOv8 incorporates an early stopping mechanism to prevent model overfitting, whereby training is terminated if no significant improvement is observed over a specified number of epochs. The “patience” parameter is indicative of the number of epochs for which no discernible progress is tolerated before training halts. In our case, “patience” was set at 50 epochs, and the “fitness” criterion was defined as a weighted combination of metrics, with (0.1 × mAP50 + 0.9 × mAP50-95) utilized to assess the performance of the CNNs model. Following 300 epochs of training, YOLOv8s exhibited its best performance when the model depth was a multiple of 0.33, width was a multiple of 0.5, and the maximum number of channels was set to 1024. The results of these experiments are presented in Table 3, with YOLOv8s outperforming the other three models, achieving the highest mAP50 at 74% and the highest fitness score at 37.64%. Consequently, YOLOv8s was selected as the baseline model for further analysis.

### 5.3. Performance Comparison of Different Loss Functions 

To assess the superiority of the MPD-IoU loss function in the recognition of the entire crack dataset, we conducted a comparative analysis involving three different loss functions: C-IoU, W-IoU, and MPD-IoU, all within the YOLOv8s framework. These experiments entailed an examination of three distinct bounding box regression loss functions, while maintaining identical network structures across all three schemes, with the sole variation being the choice of loss function. Furthermore, all three schemes were trained using the same parameters. In the context of image segmentation, “recall” signifies the model’s capability to accurately detect target objects, representing the proportion of pixels effectively identified by the model as true targets. Conversely, “precision” quantifies the proportion of pixels within the model’s segmentation output that are accurately classified as target objects. The relationship between recall and precision in image segmentation often involves a trade-off, necessitating the introduction of the F1-score to strike a balance between these two metrics. Enhancing the model’s sensitivity to target objects (improving recall) may lead to the inclusion of more target regions in the segmentation results but can also result in an increase in false positives. Hence, reducing false positives (enhancing precision) may lower recall as the model adopts a more conservative approach, becoming less inclined to classify boundary regions as target objects.

Table 4 reports a performance comparative analysis based on the test dataset. Notably, in the specific context of bridge crack detection, the ramifications of false negatives can be exceedingly detrimental, potentially causing significant financial losses and even posing risks to human safety. Therefore, when their F1-scores are approximately equal, prioritizing recall over precision is advisable during bridge inspection. As a result, the “YOLOv8s + AFPN + MPDIoU” combination emerges as the preferred choice. Furthermore, it exhibits improvements of 1.3% in terms of mAP50 and mAP75 when compared to “YOLOv8s + AFPN + CIOU” on the test dataset, as well as enhancements of 0.3% and 0.8% in mAP50 and mAP75 compared to “YOLOv8s + AFPN + WIOU” on the same test dataset. 

### 5.4. Performance Comparison of Instance Segmentation Models 

To assess the effectiveness of the proposed YOLOv8s + AFPN + MPDIoU model, we conducted a comprehensive comparative analysis alongside three established instance segmentation models, with a focus on key performance metrics including precision, recall, F1-score, mAP50, and mAP75. Results of the conducted performance comparative analysis are given in Table 5. They highlight the superiority of the YOLOv8s + AFPN + MPDIoU model, which achieved a precision rate of 90.7%, a recall of 70.4%, an F1-score of 79.27%, mAP50 of 75.3%, and mAP75 of 74.80%. In comparison to the YOLOv8s model, our proposed model exhibited a minor decrease in precision but demonstrated improvements in other performance indicators, with recall, F1-score, mAP50, and mAP75 increasing by 1.8%, 0.46%, 1.3%, and 1.4%, respectively. Regarding model complexity, the proposed model has a relatively compact size, requiring only 22.7 M of computer storage, making it larger only than the YOLOv5s-seg model. This compact size facilitates the installation of the model on portable equipment for practical bridge inspections. Notably, Mask-RCNN, being a two-stage instance segmentation model with the most parameters in comparison to other models in this study, consumes the highest amount of computer storage at 335 M. Additionally, for the YOLOv8-seg model, an increase in channel width and depth corresponds to an escalation in model size, with YOLOv8s-seg at 22.7 M, YOLOv8m-seg at 67.8 M, and YOLOv8l-seg at 151 M. Moreover, the model size also influences training and inference times. The most complex model, Mask-RCNN, requires the longest training time (approximately 11.5 h) and inference time (36 ms per image). In contrast, our model, YOLOv8s + AFPN + MPDIoU, takes the second place in terms of model complexity and requires 12.2 ms to recognize bridge cracks in an image. Consequently, YOLOv8s + AFPN + MPDIoU emerges as the optimal choice, effectively balancing precision, time efficiency, and ease of installation.

### 5.5. Crack Measurement Performance 

**Figure 14:**
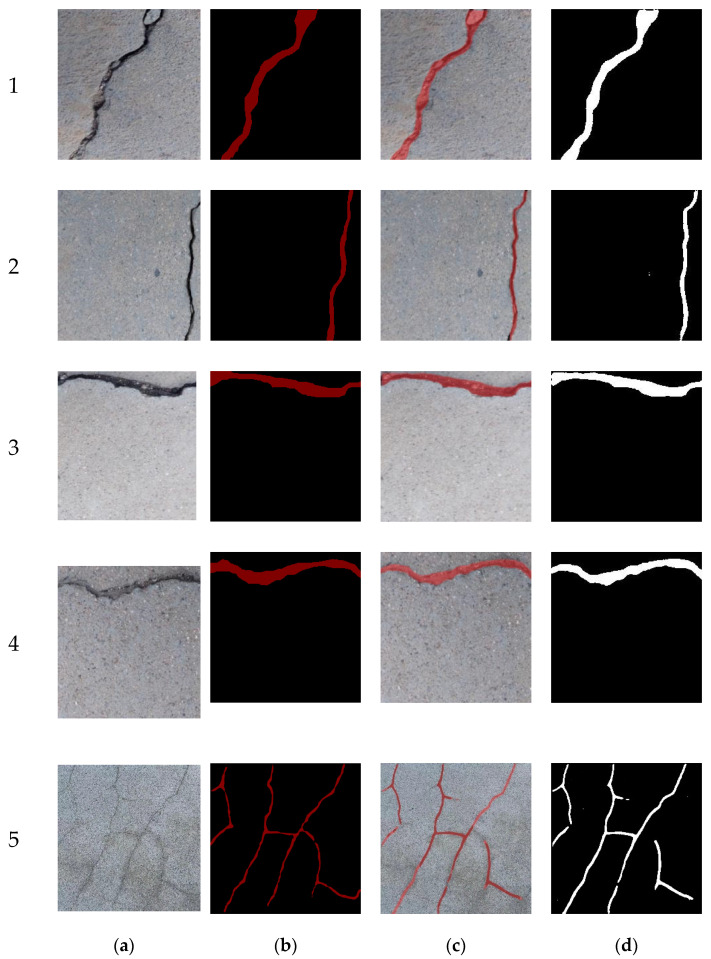
Samples of the crack measurement. (**a**) Original crack images; (**b**) manually segmented crack images; (**c**) segmented crack images with original background; and (**d**) segmented crack images. 1: Diagonal crack, 2–4: Linear cracks and 5: eticular crack.

In the final section, the proposed model effectively segmented various types of cracks in diverse structural and environmental contexts (see Figure 14). To further augment its functionality, we introduced a width measurement module designed to quantitatively assess the segmentation outcomes. While the geometric characteristics of cracks are typically described in pixel terms, determining the actual width of a crack necessitates the conversion of pixel measurements into internationally recognized standard units of measurement. Furthermore, potential correction factors, such as accounting for the angle between the camera and the photographed surface, must be taken into consideration to ensure precise width measurements. These considerations will be a focal point in our future research endeavors, aimed at refining and enhancing the model’s measurement capabilities. As observed in Table 6, it is clear that the discrepancy between the actual values and the segmentation results is quite minimal for the majority of diagonal and transverse cracks. However, owing to their intricate nature, the model frequently generates misidentifications and incorrect predictions when dealing with reticular bridges. This highlights the imperative need for further refinement in enhancing the model’s accuracy in the detection and prediction of reticular cracks within bridge structures. Tackling this specific challenge will be a primary focus of our forthcoming research and development endeavors, with the ultimate aim of bolstering the model’s capabilities and ensuring precise segmentation outcomes across all types of cracks, even within complex structures such as reticular bridges. 

**Figure 15:**
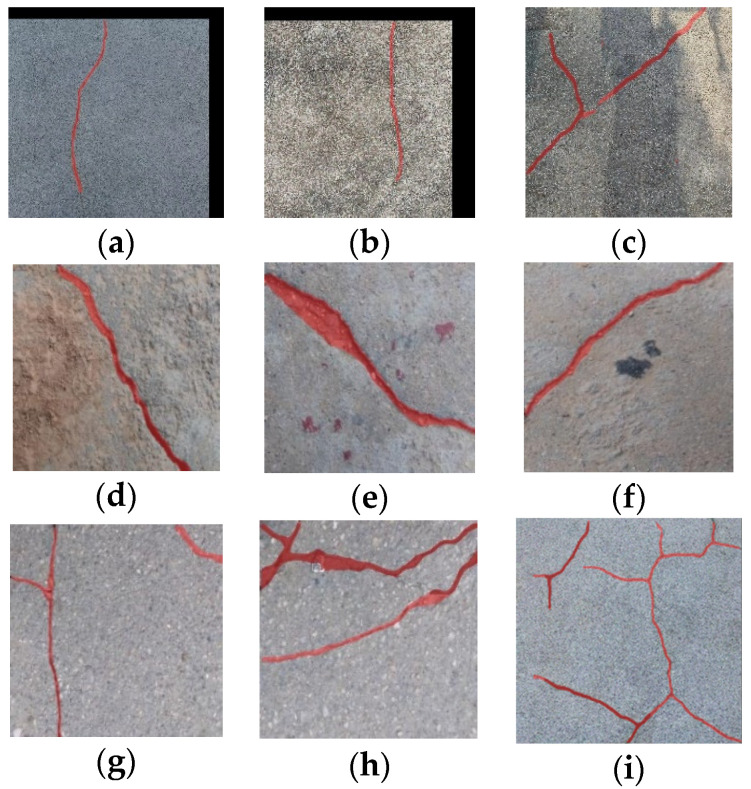
Samples of the segmented images with (**a**–**c**) different lighting conditions, (**d**–**f**) stains, and (**g**–**i**) reticular cracks.

Figure 15 expounds another portion of segmented images by the developed YOLOv8-AFPN-MPD-IoU model. Through comprehensive analysis of the results from the test dataset, the proposed model demonstrated robust generalization capabilities across various environmental conditions. Specifically, the model effectively identified cracks under different lighting scenarios (e.g., low light, direct sunlight) and weather conditions (e.g., cloudy day, clear skies), as illustrated in Figure 15a–c. Moreover, the model exhibited strong anti-interference capabilities, accurately distinguishing cracks from external objects such as water stains, graffiti, and shadows, as shown in Figure 15d–f. The model performed well in detecting single horizontal and vertical cracks, as well as simple branched cracks (Figure 15g–i). Overall, these results indicate that the model can generalize well across various environmental conditions, enhancing its applicability in real-world scenarios, where such variability is common.

## 6. Conclusions 

This study introduced a novel computer vision model aimed at effectively segmenting bridge cracks and deriving precise geographic information from the segmented crack images at the pixel level. The traditional method of human visual inspection is not only time-consuming and subjective but also poses risks such as falls and injuries. Although existing object detection models have mitigated these issues, they primarily rely on qualitative analyses for crack evaluation. In contrast, our proposed model offers a groundbreaking quantitative analysis, empowering bridge inspectors to make informed decisions. The superiority of the developed YOLOv8s + AFPN + MPDIoU model lies in the following:

1. Balanced accuracy and speed: By employing a one-stage instance model, we strike a crucial balance between segmentation accuracy and processing speed. This approach avoids the limitations of two-stage methods that may not comprehensively capture the interplay between detection and segmentation. Multi-stage methods, often associated with extended processing times, are circumvented, enabling real-time segmentation.
2. Innovative feature fusion: The incorporation of an asymptotic feature pyramid network in the YOLOv8-seg model replaces conventional top-down or bottom-up feature pyramid networks. This innovative choice addresses the loss and deterioration of feature information between non-adjacent layers, facilitating improved feature fusion and enhancing the overall model performance.
3. Specialized loss function: The introduction of the minimum point distance-IoU loss function tackles issues arising when the predicted bounding box possesses the same aspect ratio as the ground-truth bounding box but exhibits distinct width and height values. This tailored loss function ensures a more accurate and reliable model.
4. Quantitative measurement method: The combination of the middle aisle transformation method and Euclidean distance method to calculate the length and width of bridge cracks in segmented images provides a quantitative basis for maintenance suggestions. This method enhances the precision of crack assessment.

Furthermore, in terms of the loss function selection, the MPD-IoU loss function emerges as the optimal choice, achieving the highest recall score. Given that every crack poses a potential threat to bridge health, this selection is crucial. Comparative analysis with other instance models reveals that the YOLOv8s + AFPN + MPDIoU model outperforms others in terms of model size, inference time, and mAP50, making it the optimal choice for practical industry applications. Additionally, the evaluation of max width and length errors between manual inspection and segmented crack results indicates a minimal discrepancy of around 5%, significantly smaller than the errors associated with visual inspection. This underscores the reliability and precision of our proposed model in assessing bridge cracks. In summary, our computer vision model stands out as an innovative, efficient, and reliable solution for bridge inspection and maintenance in the industry.

## Figures and Tables

**Table 1:** Partitioning of bridge crack dataset.

Task	Dataset	Training	Validation	Testing	Total Number
Segmentation	BSD	952	136	272	1360
	ours	168	24	48	240
	New dataset	1120	160	320	1600

**Table 2:** Hardware and software specifications.

Hardware	Information	Software	Information
GPUs	GeForce RTX 3090	Operating System	Windows 10 (Version 23H2)
CPU	Intel(R) Core (TM) i9-10900 CPU @ 2.80 GHz	Deep Learning Framework	PyTorch
RAM	22.6 GB	CUDA Version	11.7
		Python Version	3.8

**Table 3:** Performance comparison of YOLOv8 architectures based on test dataset.

Model	Epoch	Size	Running Time	P	R	mAP50	mAP75	Map50-95	F1	Model Fitness
YOLOv8n	300	6.43 M	4.028 h	88.80%	71.10%	72.60%	71.60%	32.90%	78.97%	0.3687
YOLOv8s	300	22.7 M	3.385 h	92.60%	68.60%	74.00%	73.40%	33.60%	78.81%	0.3764
YOLOv8m	300	67.8 M	3.783 h	90.20%	68.10%	71.30%	70.40%	32.40%	77.61%	0.3629
YOLOv8l	300	151 M	6.164 h	88.90%	70.20%	73.70%	73.20%	33.50%	78.45%	0.3752

**Table 4:** Performance comparison of loss functions based on testing dataset.

Model	Epoch	Model Size	Running Time	P	R	mAP50	mAP75	F1
YOLOv8s + AFPN + CIoU	300	23.7 MB	3.596 h	0.935	0.694	0.74	0.735	79.69%
YOLOv8s + AFPN + MPDIoU	300	22.7 MB	3.661 h	0.907	0.704	0.753	0.748	79.27%
YOLOv8s + AFPN + WIoU	300	23.7 MB	3.575 h	0.936	0.677	0.75	0.74	78.57%

**Table 5:** Performance comparison of instance segmentation models based on testing dataset.

Model	Epoch	Model Size	Inference Time	Running Time	MASK				
					P	R	F1	mAP50	mAP75
YOLOv5s-seg	282	18.79 M	11.5 ms	2.397 h	90.50%	68.80%	78.17%	72.30%	70.60%
YOLOv8s-seg	300	22.7 M	8.9 ms	3.385 h	92.60%	68.60%	78.81%	74.00%	73.40%
YOLOv8m-seg	300	67.8 M	12.5 ms	3.783 h	90.20%	68.10%	77.61%	71.30%	70.40%
YOLOv8l-seg	300	151 M	12.5 ms	6.164 h	88.90%	70.20%	78.45%	73.70%	73.20%
Mask-RCNN	300	335 M	36 ms	11.5 h	49.82%	26.10%	34.25%	49.80%	7.60%
YOLOv8s + AFPN + MPDIoU (ours)	300	22.7 MB	12.2 ms	3.661 h	90.70%	70.40%	79.27%	75.30%	74.80%

**Table 6:** Comparison of manually measured and segmentation.

		Ground Truth		Segmentation		Error	
Image Number	Type	Max Length	Max Width	Max Length	Max Width	Max Length	Max Width
1	Diagonal	254.00	23.75	259.00	24.83	−1.97%	−4.56%
2	Diagonal	225.00	7.64	229.00	7.64	−1.78%	0.00%
3	Transverse	230.00	21.08	225.00	21.01	2.17%	0.33%
4	Transverse	227.00	19.93	228.00	18.02	−0.44%	5.31%
5	Reticular	4568.00	40.11	4513.00	39.03	1.20%	2.70%

## Data Availability

Some or all the data that support the findings of this research study are available upon request from the corresponding author.
